# Impacts of cobalt and zinc on improving peanuts nutrient uptake, yield and irrigation water use efficiency under different irrigation levels

**DOI:** 10.1038/s41598-024-56898-2

**Published:** 2024-03-26

**Authors:** Ayman M. S. Elshamly, Saad M. A. Nassar

**Affiliations:** 1 Water Studies and Research Complex, National Water Research Center, Cairo, Egypt; 2 Department of Genetic Resources, Desert Research Center, El-Matareya, Cairo, Egypt

**Keywords:** *Arachis hypogaea* L., Nutrients, Water regime, Environmental impact, Plant sciences

## Abstract

The knowledge of proper fertigation across various irrigation levels is necessary for maximizing peanut yield and irrigation use efficiency in arid areas, and it also can effectively alleviate the risk of nutrient deficiency induced by water stress. This study evaluated the effectiveness of cobalt combined with two zinc application methods on peanut nutrient uptake, yield, and irrigation water use efficiency across varying irrigation levels. A split-split plot experiment was carried out in 2021 and 2022. Three peanut gross water requirement (GWR) levels (100%, 80%, and 60%) were designated for main plots. Subplots featured plants treated with either 0 or 7.5 mg L^−1^ of cobalt. The sub-sub plots assessed chelated zinc effects at rates of 0 and 2 g L^−1^ via foliar and soil applications. In comparison to the control (100% GWR), nutrient uptake decreased, with sodium being the exception, and there was an increase in soil pH at 60% GWR. The results showed also significant reductions in yield and water use by approximately 60.3% and 38.1%, respectively. At this irrigation level, applying zinc via soil, either alone or combined with cobalt, led to significant yield increases of 89.7% and 191.3% relative to the control. Also, it’s crucial to note that cobalt application negatively affected iron and copper at 60% GWR, but this impact was lessened with soil-applied zinc. Hence, under a similar circumstance, treating stressed peanut plants with additional foliar applications of iron + copper and applying zinc via soil, could enhance nutrient uptake and improve yield. On the other hand, at 80% GWR, a combination of foliar-applied zinc and cobalt, had a tremendous impact on the absorption of (nitrogen, phosphorus, magnesium, and zinc), resulting in enhanced agronomic traits and decreased water losses. Additionally, at this irrigation level, foliar zinc application alone yielded a 32.4% increase compared to the 80% GWR control. When combined with cobalt, there was a 70.0% surge in water use. Based on this knowledge, the study suggests using 80% GWR and treating peanut plants with a combination of foliar-applied zinc and cobalt. This strategy aids plants in countering the adverse effects of water stress, ultimately leading to enhanced yield and irrigation water use efficiency.

## Introduction

Globally, peanut crops (*Arachis hypogaea*) are regarded as one of the primary oilseed crops and summer food legumes in arid and semi-arid regions^1,2^. Africa alone accounts for 57% of the world’s peanut area, covering 29.6 million hectares, and contributes 34% to the global peanut production of 48 million tons^3,4^. Therefore, extensive research aimed at enhancing peanut yield and water use efficiency under stressful conditions^5,6^, would have a significant impact on large-scale cultivation and positively affect global production.

In arid regions, water stress poses significant limitations to worldwide agricultural production^7,8^. Its adverse effects on agronomic traits and the availability of macronutrients and micronutrients lead to a considerable reduction in yield^5^. Consequently, in the face of impending water scarcity, there is a need for further studies on optimal management of irrigation water techniques^6,9^.

Egypt has a total land area of 100 million hectares (Mha), out of which only 3.78 Mha are considered cultivated, while the rest remains desert. The country heavily relies on the River Nile, which provides 55.5 billion cubic meters of water, falling short of the demand of 72.5 billion cubic meters^10^. The desertic lands in Egypt suffer from nutrient deficiency, limited irrigation water, higher soil pH, low water and nutrient retention capacity, low organic matter content, and reduced microbial communities^11^. Cultivating leguminous plants like peanuts in these soils not only improves organic levels but also lowers their alkaline pH. The significance of this pH change lies in its strong influence on nutrient availability^12^. Additionally, peanut plants possess unique abilities to enhance nutrient bioavailability in the soil through increased root exudates, allowing them to adjust soil pH and control the balance of cation and anion nutrients, as observed in various previous studies^13–17^.

Considering the aforementioned information, it can be hypothesized that cultivating peanut plants in these environments has the potential to improve soil quality and yield. However, it is crucial to explore whether supplying these plants with appropriate applications to aid in improving plant development and reducing water loss would be more effective under such water-stressed conditions.

Cobalt (Co), a transitional element, plays a crucial role as an essential component in numerous enzymes and coenzymes^18^. Low concentrations of Co have unique influences that can enhance both nutritional status and plant tolerance. It interacts with zinc (Zn) to maintain cell homeostasis, and its supplementation improves nitrogen fixation, thus promoting legume growth^19,20^. Applying Co to legume crops increases root and microbiome exudates in the rhizosphere, leading to soil acidification^21^. However, like some micronutrients, plants respond positively to low concentrations of Co in the soil, which promotes plant growth, yield, decreases water loss, and affects the content of macronutrients and micronutrients^5^, Gad et al.^22^^,^^23^, while higher concentrations induce phytotoxicity^24^. Despite several studies demonstrating the benefits of low Co on plant growth and nutrient uptake under normal conditions, there is a lack of research investigating its impacts on nutritional uptake status when peanuts are exposed to prolonged water stress conditions, which may result in an increase in Co molecules around the roots and ultimately affect plant development and yield.

To counteract the deleterious impacts of unfavorable conditions, improving water status and physiological processes within plants are essential. In this regard, Zn application is commonly used to overcome the drastic impacts of water stress^25^. Where Zn serves as a cofactor for different enzymes involved in various physiological processes. It is an essential micronutrient required for the growth and development of crop plants in small amounts (15–20 mg kg^−1^)^26^. Globally, due to its lower availability, Zn is considered the most limiting micronutrient in crop yield^27^. While the behavior and impacts of Zn under water stress are not fully understood, it has been suggested to enhance water use efficiency^28^. Numerous studies have demonstrated that Zn application plays a vital role in counteracting water stress impacts by enhancing water relations for plants, stabilizing cell membranes, promoting chlorophyll formation, accumulating osmolytes, regulating carbohydrate metabolism, enhancing protein synthesis, controlling stomatal regulation and photosynthesis, and consequently improving plant performance significantly^29–31^.

To fully capitalize on the multiple benefits of both Co and Zn applications, further research is required. Using low concentrations of Co, some negative impacts on the uptake of certain nutrients, particularly micronutrients, have been observed. For example, Liu^32^ demonstrated that increasing Co content led to a significant decrease in Zn uptake, especially under increasing water stress intensity. However, previous studies have shown that Co has a favorable effect on nutrient status, including Zn^5,22,24^. To explain this, Khrustalev et al.^33^ found that Co is commonly bound by cation trap sites, which are normally occupied by Cu, Fe, Mn, and Zn. Thus, higher Co concentration has the ability to replace these ions in the active sites^20^. Elshamly^5^ noted that the relationship between micronutrient uptake as a consequence of Co usage fluctuated, which was attributed to the irrigation pattern implemented.

Based on the aforementioned, it can be hypothesized that as water stress intensity increases, Zn uptake levels interact negatively with Co and other micronutrients, resulting in restricted root growth, reduced yield, and decreased Zn absorption due to high pH values^34–36^**.** Therefore, applying Zn in combination with Co under these circumstances in an appropriate method is crucial for crop yield and water use efficiency. Previous studies have varied in determining the optimal method of applying Zn alone. Hussain et al.^37^, indicated that foliar Zn applications led to marked increases in yield compared to soil applications, while other studies mentioned that applying Zn through either method enhanced growth and yield^38^. On the other hand, injecting Co through irrigation water has been preferred^22,24^ for improving roots and microbiome exudate quantities, which results in soil acidification^5^. Thus, foliar utilization is hypothesized to be an effective method of applying Zn in combination with Co under these conditions. Nevertheless, as mentioned above, the reactions of each examined treatment (peanuts, Co, and Zn) vary depending on the intensity of irrigation level and the effectiveness of these applications. Therefore, to test the validity of this hypothesis, studying and understanding the overall influences that appear under different water conditions is crucial.

Due to limitations in studies investigating the role of Co and Zn in enhancing legume yield under different water conditions, this experiment was conducted to test their impacts as sole and combined applications on improving the yield and irrigation water use efficiency of peanuts. Additionally, the study aims to identify the preferred method of applying Zn, either through foliar spraying or soil applications, in combination with Co when peanut plants are exposed to different water levels.

## Materials and methods

### Site description

At the experimental farm of water studies and research station, National Water Research Center (NWRC), Abu Simbel City, Egypt, the experiment study was conducted during two successive summer seasons of 2021 and 2022. Toshka region has an arid climate with an annual precipitation recorded zero throughout the two growing seasons of 2021 and 2022. During the study period, the mean maximum temperature values ranged from 97.7 to 109.6 °F in the first season and from 97.2 to 108.9 °F in the second season. The mean minimum temperature values ranged from 69.8 to 77.7 °F in the first season and from 70.3 to 80.1 °F in the second season. Monthly mean temperature, wind speed, relative humidity, solar radiation, and precipitation are presented in **(**Table 1). Chemical analysis of irrigation water during the growing seasons was conducted **(**Table 2). According to Badr and Al-Naeem^39^, pH and total dissolved solids (TDS) values were measured directly in the field during sample collection. Standard procedures for water and wastewater guidelines were adopted and followed for conducting the laboratory analysis of irrigation water samples^40^. As described by Hanrahan et al.^41^, the sulphate (SO_4_^2−^) was analyzed in irrigation water using a UV–Visible Spectrophotometer, by using the turbidimetric method. The concentrations of chloride (Cl^−^), Bicarbonate (HCO_3_^−^), calcium (Ca^2+^), and magnesium (Mg^2+^) were analyzed using trimetric methods, as outlined by Adams^42^. The flame emission photometric was used for the analysis of potassium (K^+^) and sodium (Na^+^) in irrigation water^40^. The initial (physical and chemical) properties of the soil were measured during both seasons **(**Table 3**)**. From each plot, soil samples were collected at two depths (i.e., 0–30 cm and 30–60 cm) using a 2.5 cm diameter spiral auger. From each plot, three sub-samples were taken to make a composite sample per plot. Soil particle size distribution was measured by the hydrometer method, as according to Estefan et al.^43^. In accordance with the methods of USDA Soil Survey Staff^44^, the selected soil has a sandy texture. The other physical and chemical properties of the soil were determined by following Estefan et al.^43^. The available potassium present in soils was determined by using the flame photometer method. The pH and TDS were also tested before planting the crop using a pH meter and an EC meter. Soil organic matter was measured with the wet digestion method. Furthermore, total carbonates were measured as CaCO_3_ using Collin’s calcimeter. 1 N ammonium acetate solution (NH_4_AC) at pH = 7.0 (W/V) was used to extract the soluble cations of (Ca^2+^, Mg^2+^, K^+^, and Na^+^), then K^+^ and Na^+^ were determined using flame photometry. Both Ca^2+^ and Mg^2+^ were determined by the titration method using EDTA. Also, the soluble anions of HCO_3_^−^ and Cl^−^, were measured by the titration method, while sulfate (SO_4_^2−^) was determined by the turbidimetric method using a spectrophotometer at 470 nm wavelength.

**Table 1:** Weather data from the experimental site during 2021/2022 growing seasons.

		Temperature (°F)		Relative humidity (%)		Wind speed (Km hour^−1^)	Solar radiation (MJ m^−2^)	Precipitation (mm)
		Max	Min	Max	Min			
June	2021	107.6 ± 0.20	77.2 ± 0.21	30.1 ± 0.20	3.4 ± 0.21	11.9 ± 0.20	27.0 ± 0.21	0
	2022	106.2 ± 0.22	75.6 ± 0.22	29.1 ± 0.21	4.9 ± 0.20	12.2 ± 0.20	26.8 ± 0.20	0
July	2021	109.6 ± 0.21	76.1 ± 0.20	28.8 ± 0.22	5.3 ± 0.25	9.0 ± 0.24	27.0 ± 0.20	0
	2022	108.9 ± 0.20	75.7 ± 0.21	28.1 ± 0.21	3.3 ± 0.22	7.9 ± 0.24	26.2 ± 0.21	0
August	2021	108.1 ± 0.21	77.7 ± 0.19	31.3 ± 0.22	5.8 ± 0.22	9.7 ± 0.22	20.5 ± 0.22	0
	2022	108.5 ± 0.21	78.8 ± 0.19	33.3 ± 0.22	5.6 ± 0.22	9.4 ± 0.22	21.2 ± 0.21	0
September	2021	106.7 ± 0.19	75.9 ± 0.20	37.1 ± 0.21	9.4 ± 0.21	11.5 ± 0.24	18.0 ± 0.19	0
	2022	105.3 ± 0.20	80.1 ± 0.21	37.5 ± 0.22	9.3 ± 0.22	11.2 ± 0.21	18.6 ± 0.21	0
October	2021	97.7 ± 0.20	69.8 ± 0.21	43.0 ± 0.21	12.6 ± 0.29	13.7 ± 0.20	16.1 ± 0.18	0
	2022	97.2 ± 0.20	70.3 ± 0.21	48.2 ± 0.23	16.1 ± 0.25	13.3 ± 0.21	16.0 ± 0.20	0

Max maximum temperature, Min minimum temperature, and mm millimeter. The meteorological data were obtained from Toshka Agrometeorological Station, Egypt. Values are the mean of replicates ± standard errors.

**Table 2:** Water chemical properties at the experimental site, Egypt during the growing seasons 2021–2022.

Parameter	Unit	Value	References
pH		6.30 ± 0.70	Estefan et al.^43^
TDS	mg L^−1^	640.5 ± 0.72	
HCO_3_^−^	mg L^−1^	68.5 ± 0.73	
Calcium cations (Ca^2+^)	mg L^−1^	65.2 ± 0.70	
Magnesium cations (Mg^2+^)	mg L^−1^	16.2 ± 0.71	
Sodium cations (Na^+^)	mg L^−1^	118.2 ± 0.70	
Potassium cations (K^+^)	mg L^−1^	4.8 ± 2.41	
Chloride anions (Cl^−^)	mg L^−1^	111.7 ± 0.70	
Sulfate anions (SO_4_^2−^)	mg L^−1^	245.0 ± 0.71	

Each value represents the mean of replications ± standard errors.

TDS, total dissolved solids.

**Table 3:** The initial (physical and chemical) properties of the soil at the experimental site, Egypt in both 2021 and 2022 growing summer seasons (data pooled over both seasons).

Parameter	Unit	Soil depth (cm)		Reference
		0–30	30–60	
Mechanical analysis				Estefan et al.^43^
Sand	% by weight	90.48 ± 0.72	91.36 ± 0.71	
Silt	% by weight	2.56 ± 0.70	2.27 ± 0.71	
Clay	% by weight	6.46 ± 0.70	5.57 ± 0.70	
Texture	Sand			
Chemical analysis				
pH		6.84 ± 0.71	7.14 ± 0.70	
TDS	meq L^−1^	420 ± 0.73	245 ± 0.71	
CaCO_3_	% by weight	8.25 ± 0.70	7.85 ± 0.72	
Calcium cations (Ca^2+^)	meq L^−1^	1.7 ± 0.74	1.1 ± 0.71	
Magnesium cations (Mg^2+^)	meq L^−1^	0.2 ± 0.80	0.3 ± 0.75	
Sodium cations (Na^+^)	meq L^−1^	4.2 ± 0.71	2.0 ± 0.70	
Potassium cations (K^+^)	meq L^−1^	0.1 ± 0.71	0.2 ± 0.70	
Chloride anions (Cl^−^)	meq L^−1^	2.8 ± 0.72	1.7 ± 0.71	
Bicarbonate anions (HCO_3_^−^)	meq L^−1^	0.9 ± 1.10	0.6 ± 1.20	
Sulfate anions (SO_4_^2−^)	meq L^−1^	3.1 ± 0.74	1.8 ± 0.72	
Potassium available	mg L^−1^	125 ± 0.70	88 ± 0.71	
Organic matter	% by weight	0.3 ± 2.0	0.3 ± 2.12	

Each value represents the mean of replications ± standard errors.

### Experimental details

In order to achieve the objective of the current study, the field experiment was laid out in a split-split plot design using randomized complete blocks arrangement with three replications. Irrigation water schemes were assigned to the main plots, while Co and Zn treatments were assigned to the sub and sub-sub plots, respectively. Irrigation amounts equaled 100% (representing no water stress and denoted by 100% GWR), 80% (denoted by 80% GWR), and 60% (denoted by 60% GWR), were levels of irrigation. A drip irrigation system was used to irrigate peanut plants. Each plot was equipped with a manometer valve to keep the operating pressure at 1 bar. Also, there was a water emitter used to control the quantity of the targeted irrigation water. To avoid the seepage impact, 3 m width buffer zones were surrounded the experimental units, and maintained throughout the two growing seasons. On the other hand, Co application in a form (CoSO_4_·7H_2_O) was assigned to the subplots at rates of (Co = 0 mg L^−1^ and Co = 7.5 mg L^−1^). This product was purchased from Sigma-Aldrich Company. The subplots were also equipped with a valve; thus, one dose of Co was injected in the drip irrigation system by a venture injector after 30 days from emergence. On the other side, sub-sub plots were divided into three parts, then chelated Zn applications in a form (EDTA) was used at rates of: 0 (denoted, control- sprayed with distilled water), 2 g L^−1^, which was applied as foliar applications after 60 and 75 days of sowing date, denoted Znfol, chelated Zn applied as soil applications at rates of 10 (kg ha^−1^), which were injected in the drip irrigation system twice after 30 and 60 days of sowing date in two equal doses, denoted Zninj. The control treatment (0 mg L^−1^) was sprayed with an equal amount of distilled water for comparison. The sub-sub plot area was 10.0 m long × 5 m width. Accordingly, the experimental work involved 54 plots {3 irrigation levels × 2 Co treatments × 3 Zn treatments × 3 replicates, as shown in Figure S1. Peanut seeds (Cv. Giza 6) were obtained from oil crops department, field crops institute, agricultural research center, Egypt. This cultivar is recommended as a highly yielding commercial cultivar. Furthermore, the cultivar and methods in the current study were complied with international, national, and institutional guidelines and legislation. Prior to sowing, peanut seeds were inoculated with appropriate *Rhizobium spp*, which purchased from the central laboratory of organic agriculture, agricultural research center, Egypt. to increase biological nitrogen fixation. The specific strain, namely *Bradyrhizobium japonicum*. A recommended peanut seeds rate of 108 kg per ha was used. Peanut seeds were planted on June 12nd 2021 and June 15th 2022, respectively. Two seeds were sown directly in hills by hand and maintained a distance between hills at 20 cm and between rows at 75 cm. All treatments were fertilized upon the recent recommendations the Ministry of Agriculture and land reclamation in Egypt. The recommended phosphorus rate of 480 kg ha^−1^, added as calcium super phosphate (15.5% P_2_O_5_), was applied to the soil during field preparation through one application. Meanwhile, nitrogen in the form of ammonium nitrate (33.5% N), was added as 36 kg N ha^−1^, which was added with irrigations in two equal doses; 3 and 6 weeks after sowing. Potassium fertilizations was applied in the form of potassium sulphate (48% K_2_O), that was added at 240 kg K ha^−1^ in two equal doses (on 21 and 35 days after sowing). Along the experimental periods, no serious incidence of diseases was observed. Also, weeds in the experimental plots were controlled manually with all the crop components.

### Calculations of water requirements

The applied irrigation water amounts at each irrigation levels were based on ETo and ETc, which was calculated as shown in Eq. (1 and 2)

Daily weather data were obtained from Toshka agrometeorological station, to calculate reference evapotranspiration (ETo). Reference evapotranspiration was calculated according to Priestley-Taylor equation which was recommended to calculate ETo for peanut in Toshka region^45^, using the mean climatic data from 2021 to 2022. Priestly -Taylor equation (Eq. 1), was presented by Nikolaou et al.^46^ as follows:

1 $$ {\text{ETo}} = \propto \frac{{{\Delta }}}{{{\Delta } + {\gamma }}} \frac{{\left( {{\text{Rn}} - {\text{G}}} \right){ }}}{{{\lambda }}} $$

where ∝  = evaporative coefficient. Δ = the slope of the saturated vapor pressure curve (kPa °C^−1^). γ = the psychrometric constant (kPa °C^−1^). Rn = the net radiation (MJ m^−2^ d^−1^). λ = the latent heat of vaporization (MJ kg^−1^). G = the soil heat flux (MJ m^−2^ d^−1^).

While, peanut crop evapotranspiration was determined according to Deepa et al.^47^ as the following equation (Eq. 2):

2 $$ {\text{ETcrop}} = \left( {{\text{ETo}} \times {\text{Kc}}\;{\text{stage}}} \right) $$

where ETcrop = Crop evapotranspiration of peanut (mm day^−1^). ETo = Reference evapotranspiration (mm day^−1^). Kc = Crop coefficient (was equaled 0.60, 0.76, 0.88, 0.78, and 0.63 for Kc_initial_, Kc_development_, Kc_mid_, Kc_late_, and Kc_harvest_ according to Zayton et al.^48^.

Then, to calculate the amount of water applied at each irrigation for (100%) GWR, the method of Al-Omran et al.^49^ & El Namas et al.^50^ was adopted as follows (Eq. 3);

3 $$ {\text{GWR}} = \frac{{{\text{ETc}} \times Se}}{{{\text{E}}a \times \left( {1 - LR} \right)}} \times 10 $$

where GWR = The gross water requirement (m^−3^ ha^−1^). Se = The percentage of evapotranspiration area. LR = Leaching requirement 10%. Ea = Irrigation system efficiency%. Etc = Crop evapotranspiration.

Then the of water applied amounts of (80%) GWR and (60%) GWR treatments were proportionally obtained from the (100%) GWR. Accordingly, the amounts of GWR were 9235, 7645 and 6055 for (100%) GWR, (80%) GWR, (60%) GWR, respectively.

#### Yield and yield components

At the physiological maturity, peanut plants were manually harvested on 19^th^ and 22^nd^ October in the first and second season, respectively. Moreover, a sample of ten peanut plants was collected randomly from each sub-sub plot to record the mean of:

Root length, shoot fresh weight, number of pods plant^−1^, weight of pods plant^−1^, number of seed plant^−1^, weight of seed plant^−1^. In each experimental unit of each sub-sub plot, plants on the middle two rows were collected at harvest and air-dried to account for seeds yield, and then converted to kg ha^−1^.

#### Measurement of nutrient content in seeds and pH values in soil samples

The described method by Estefan et al.^43^ was used to prepare seeds and soil pH samples. Where sufficient amounts of dried peanut seeds were weighted and milled into a fine powder. 0.5 g of the fine powder was weighed and transferred quantitatively into a 100 mL digestion tube. Then nitrogen (N) content was measured by the Kjeldahl method, as outlined by Estefan et al.^43^. On the other hand, 1 g of the fine powder was digested and mineralized with a 10 mL mixture of concentrated nitric-perchloric solution, then K, Ca, and Mg were determined using an atomic absorption spectrophotometer. While Na was measured via flame photometer, as outlined by^43,51^. Phosphorus (P) was determined by colorimeter methods using a spectrophotometer at 410-nm wavelength (Estefan et al.^43^). The concentration of {iron (Fe), manganese (Mn), Zn, and copper (Cu)} were estimated by using atomic absorption spectroscopy and following the method described by Estefan et al.^43^. On the other side, (1 mm) of soil samples were extracted with water (soil 1:1 water), to determine soil pH at depths (0–40 cm), by using digital electrodes (digital analyzer/501, Orion research multifunctional pH meter).

#### Measurement of IWUE

According to Asres et al.^52^, IWUE values were determined as the ratio between the obtained yield and GWR value as shown in Eq. (4):

4 $$ {\text{IWUE}} = { }\left( {\frac{{\text{Y}}}{{\text{GWR }}}} \right) $$

where IWUE = Irrigation water use efficiency (kg m^−3^). Y = Yield (kg ha^−1^). GWR = The gross water requirement (m^3^ ha^−1^).

### Statistical analysis

Data in each season were statistically analyzed, and the least significant differences (LSD) were in accordance with Casella et al.^53^. Also, combined analysis was performed to analyze the interactive effects of the differences among irrigation levels, Co application, Zn methods, and their interactions across the two growing seasons on the examined variables through factorial design (two-way ANOVA) in the statistical package Costat version 6.303. The mean differences among the evaluated treatments were discriminated using the protected LSD at 0.05 probability level as per Casella et al.^53^. Different lowercase letters above error bars indicate statistically significant differences (p < 0.05).

### Ethics approval and consent to participate

This manuscript is an original paper and has not been published in other journals. The authors agreed to keep the copyright rule.

## Results

### The individual and interaction impacts of irrigation levels, Co, and Zn applications on

#### N uptake

**Figure 1:**
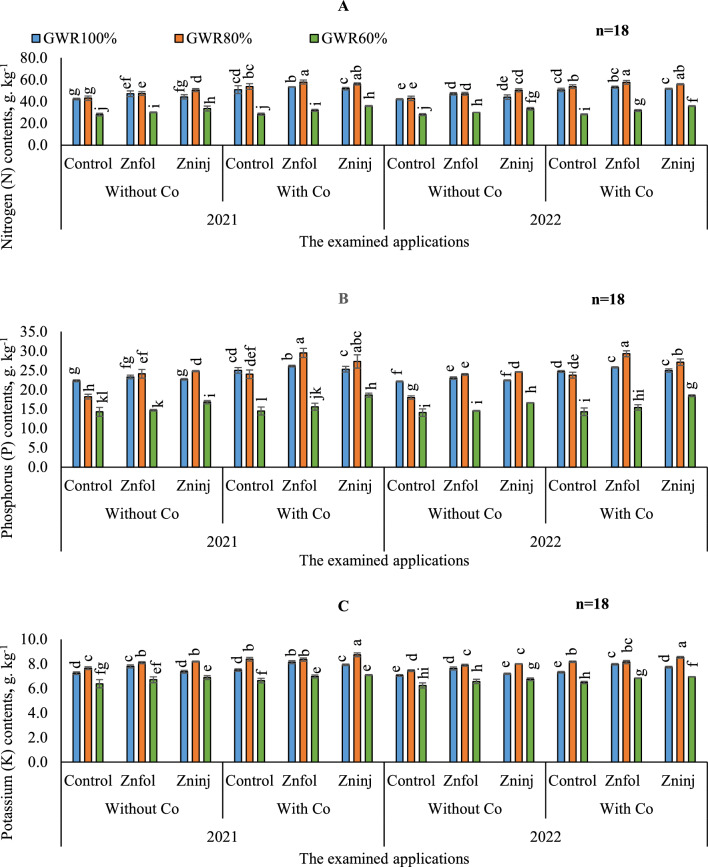
Influence of the separate or combined application of cobalt & chelated zinc under different irrigation levels during the growing seasons of 2021 and 2022 on: nitrogen (**A**), phosphorus (**B**), and potassium (**C**). Vertical bars represent ± standard error (SE) of the means. Values are means of n = 18 ± SE. Bars on the top of the columns with different letters correspond to LSD are statistically significant at p ≤ 0.05. Different lowercase letters above error bars indicate statistically significant differences (p < 0.05). Abbreviations: Control (sprayed with pure water); Znfol (foliar chelated zinc application); Zninj (soil chelated zinc application); without Co (without cobalt sulfate application); with Co (with cobalt sulfate application, 7.5 mg L^−1^); (100%) GWR (applied 100% of gross irrigation water requirements); (80%) GWR (applied 80% of gross irrigation water requirements); (60%) GWR (applied 60% of gross irrigation water requirements).

To compare the differences of N contents between the growing seasons, the results in (Fig. 1A) showed that N contents were achieved the better values when adopting (100 and 80% GWR) in the first season and (60%) GWR in the second season under different application treatments.

Based on ANOVA findings, the influence of water stress levels and examined applications (p < 0.05) on N content was significant, whereas the interaction effects between Co and Zn applications were not Table 4. According to the results in (Table 5), executing (80 or 100%) GWR levels under control treatment without applying the solitary applications of Co were statistically equivalent in terms of yielding greater N content. By comparing the solitary applications of Zn and Co, applying solitary applications of Co led to higher increases in average N contents than Zn applications under (100 and 80% GWR). However, when adopting (60%) GWR, average N content was attained the higher increases by applying solitary soil applications of Zninj. On the other hand, adopting (80%) GWR and applying combined applications of Znfol + Co achieved the maximum increase average of N content in seeds (58 g kg^−1^), where it relative to (80%) GWR under control treatment without applying the solitary application of Co, was attained increase by 25.9%.

**Table 4:** Variance analysis of the investigated parameters.

Source of variation	df	SHW	NOS	NOP	WES	WEP	RL	Y
Gross water requirements schemes (GWR)	2	*	*	*	*	*	*	*
Cobalt applications (Co)	1	*	NS	*	*	*	*	*
Zinc applications (Zn)	2	*	*	*	*	*	*	*
GWR × Co	2	*	*	*	*	*	NS	*
GWR × Zn	4	*	NS	*	*	*	NS	*
Co × Zn	2	NS	*	*	*	*	*	*
GWR × Co × Zn	24	*	NS	*	*	*	*	*

Source of variation		N	P	K	Ca	Mg	Na	Zn
Gross water requirements schemes (GWR)	2	*	*	*	*	*	*	*
Cobalt applications (Co)	1	*	*	*	*	*	*	*
Zinc applications (Zn)	2	*	*	*	*	*	*	*
GWR × Co	2	*	*	*	*	*	*	*
GWR × Zn	4	*	*	*	*	*	*	*
Co × Zn	2	NS	*	*	*	*	*	NS
GWR × Co × Zn	24	*	*	*	*	*	*	*

Source of variation		Cu	Mn	Fe	pH	IWUE		
Gross water requirements schemes (GWR)	2	*	*	*	*	*		
Cobalt applications (Co)	1	*	*	*	*	*		
Zinc applications (Zn)	2	*	*	*	*	*		
GWR × Co	2	*	*	*	NS	*		
GWR × Zn	4	*	*	*	*	*		
Co × Zn	2	*	NS	*	*	*		
GWR × Co × Zn	24	*	*	*	*	*		

SHW, shoots fresh weight; NOS, number of seeds; NOP, number of pods; WES, weight of seeds; WEP, weight of pods; RL, root length; Y, peanuts yield; N, nitrogen; P, phosphorus; K, potassium; Ca, calcium; Mg, magnesium; Na, sodium; Zn, zinc; Cu, copper; Mn, manganese; Fe, iron; pH, power of hydrogen; IWUE, irrigation water use efficiency; NS, non-significance; *significance at P ≤ 0.05.

**Table 5:** The impacts of adopting different irrigation water levels and applied chelated zinc as solitary and combined application with cobalt on the average nitrogen, phosphorus, and potassium.

Investigated parameters	Irrigation levels		
	(100%) GWR	(80%) GWR	(60%) GWR
Nitrogen (N) contents, g. kg^−1^			
Without Co			
Control	42.2 ± 0.46 g	43.0 ± 0.70 g	28.0 ± 0.42 m
Znfol	47.1 ± 1.29 f.	47.1 ± 0.88 f.	30.0 ± 0.15 kl
Zninj	44.1 ± 1.0 g	50.3 ± 0.69 de	33.4 ± 1.13 i
With Co			
Control	50.7 ± 1.64 cd	53.7 ± 1.13 bc	28.3 ± 0.31 m
Znfol	53.2 ± 0.06 c	57.7 ± 1.31 a	31.8 ± 0.49 ij
Zninj	51.7 ± 0.56 cd	55.9 ± 0.48 ab	35.9 ± 0.22 hi
Phosphorus (P) contents, g. kg^−1^			
Without Co			
Control	21.0 ± 0.08 h	18.1 ± 0.32 i	14.2 ± 0.57 l
Znfol	22.0 ± 0.23 f.	24.0 ± 0.56 e	14.6 ± 0.09 l
Zninj	22.6 ± 0.09 g	24.7 ± 0.08 d	16.7 ± 0.21 j
With Co			
Control	24.9 ± 0.37 de	23.9 ± 0.55 e	14.4 ± 0.052 l
Znfol	26.0 ± 0.11 c	29.4 ± 0.86 a	15.5 ± 0.45 k
Zninj	25.1 ± 0.38 d	27.2 ± 0.59 b	18.5 ± 0.25 i
Potassium (K) contents, g. kg^−1^			
Without Co			
Control	7.15 ± 0.057 i	7.55 ± 0.06 fg	6.31 ± 0.016 o
Znfol	7.72 ± 0.067 ef	8.0 ± 0.05 d	6.64 ± 0.12 mn
Zninj	7.28 ± 0.06 hi	8.1 ± 0.02 c	6.82 ± 0.077 lm
With Co			
Control	7.41 ± 0.052 gh	8.3 ± 0.07 b	6.56 ± 0.094 n
Znfol	8.1 ± 0.06 c	8.25 ± 0.06 b	7.02 ± 0.065 j
Zninj	7.83 ± 0.03 e	8.64 ± 0.07 a	6.81 ± 0.021 kl

The obtained values in the table are the average of the two growing seasons of 2021/2022.

Vertical bars represent ± standard error (SE) of the means. Bars on the top of the columns with different letters correspond to LSD are statistically significant at p ≤ 0.05. Different lowercase letters above error bars indicate statistically significant differences (p < 0.05). Abbreviations: Control (sprayed with pure water); Znfol (foliar chelated zinc application); Zninj (soil chelated zinc application); without Co (without cobalt sulfate application); with Co (with cobalt sulfate application, 7.5 mg L^−1^); (100%) GWR (applied 100% of gross irrigation water requirements); (80%) GWR (applied 80% of gross irrigation water requirements); (60%) GWR (applied 60% of gross irrigation water requirements).

#### P uptake

To compare the differences in P contents between the growing seasons, the results in (Fig. 1B) showed that P contents in most treatments were achieved the better values in the first season compared to the second season under different examined applications.

In general, the obtained results in (Table 5), showed that applying distilled water applications (control) under (60%) GWR was significantly equaled applying solitary applications of Co or Znfol under the same irrigation level, in attaining the lowest value of the average P contents. Compared to control (100%) GWR without applying Co, the average content of P was decreased with the adoption of (80%) GWR by 14.3% and increased by 12.5% with Co additions under the same irrigation scheme. However, the adoption of solitary application of Co under (100% GWR), improved average P content compared to control (100%) GWR without Co applications by 16.0%. While the solitary applications of Zninj were pronounced under (80% GWR) for attaining better increases in average P contents by 28%, than control (80%) GWR without Co applications, respectively. On the other side, the gained results showed that the maximum increase in the average P contents (29.0 g kg^−1^), was observed by adopting combined applications of (Znfol + Co) under (80%) GWR irrigation level.

#### K uptake

By comparing the various treatments during both growing seasons, adopting the examined treatments resulted in the better K contents in the first season than the second in most treatments, as can be seen in (Fig. 1C).

In contrast to (80%) GWR, irrigated peanut plants with (60%) GWR irrigation level showed the highest reduction in average K contents compared to control (100%) GWR, as can be seen in (Table 5). Moreover, it was noticed that the solitary applications of Znfol attained higher K contents in peanut seeds by adopting (100%) GWR. Conversely, it was shown that best average contents of K could be achieved by applying solitary application of Co under (80%) GWR (8.3 g kg^−1^). While under (60%) GWR, there were insignificant variations among the solitary examined applications. In this sense, the findings in the current study indicated that a Co supply of 7.5 mg kg^−1^ attained higher value and raised average K content under (80%) GWR by 9.6% compared to control (80%) GWR without Co application. This increase, under (100) GWR, reached almost 7.8% with Znfol supply of 2 g L^−1^ compared to control (100%) GWR without Co application. Concerning the interaction, the obtained results indicated that the adoption of (80%) GWR irrigation level and combined applications of (Zninj + Co), attained the highest values of the K contents in the seeds of peanut.

#### Ca uptake

**Figure 2:**
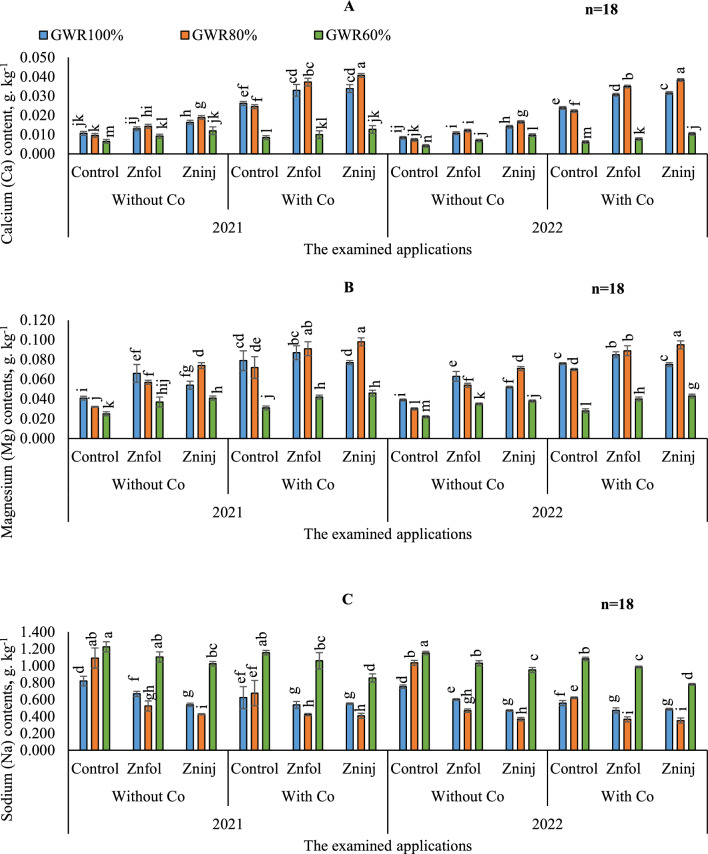
Influence of the separate or combined application of cobalt & chelated zinc under different irrigation levels during the growing seasons of 2021 and 2022 on: calcium (**A**), magnesium (**B**), and sodium (**C**). Vertical bars represent ± standard error (SE) of the means. Values are means of n = 18 ± SE. Bars on the top of the columns with different letters correspond to LSD are statistically significant at p ≤ 0.05. Different lowercase letters above error bars indicate statistically significant differences (p < 0.05). Abbreviations: Control (sprayed with pure water); Znfol (foliar chelated zinc application); Zninj (soil chelated zinc application); without Co (without cobalt sulfate application); with Co (with cobalt sulfate application, 7.5 mg L^−1^); (100%) GWR (applied 100% of gross irrigation water requirements); (80%) GWR (applied 80% of gross irrigation water requirements); (60%) GWR (applied 60% of gross irrigation water requirements).

Illustrated data in (Fig. 2A**)** showed that Ca contents were statistically better under (60% GWR) in terms of yielding the better Ca content in the first season than the second. While there were no significant differences between both seasons by adopting (100 and 80% GWR).

On the other hand, the obtained results in (Table 6**)** indicated that adopting stressful irrigation levels (80 and 60% GWR) in combination without or with Co, led to decreased Ca content. Interestingly, treatment peanut plants with Co application mixed with or even without Zn applications showed higher Ca contents. However, applying solitary application of Co under (80%) GWR, improved Ca contents better than (60%) GWR. In this sense, treating stressed plants with solitary application of Co, could help peanut plants overcome the negative impacts of water stress and attain higher Ca content. Moreover, it was found that solitary soil applications of Zninj attained higher Ca contents compared to the control treatments when adopting the different examined irrigation levels. Overall, the highest Ca content was obtained by adopting (80%) GWR irrigation level and applying combined applications of (Zninj + Co). The following highest Ca content was seen under the same irrigation level by using combined applications of (Znfol + Co).

**Table 6:** The impacts of adopting different irrigation water levels and applied chelated zinc as solitary and combined application with cobalt on the average calcium, magnesium, and sodium.

Investigated parameters	Irrigation levels		
	(100%) GWR	(80%) GWR	(60%) GWR
Calcium (Ca) contents, g kg^−1^			
Without Co			
Control	0.010 ± 0.001ij	0.008 ± 0.0005 jkl	0.005 ± 0.0005 m
Znfol	0.012 ± 0.001 h	0.013 ± 0.0003 g	0.008 ± 0.0007 jkl
Zninj	0.015 ± 0.001 f	0.018 ± 0.0007 e	0.011 ± 0.008 hi
With Co			
Control	0.025 ± 0.001d	0.023 ± 0.0007 d	0.007 ± 0.0007 kl
Znfol	0.032 ± 0.001c	0.036 ± 0.001 b	0.009 ± 0.0006 jk
Zninj	0.033 ± 0.001c	0.040 ± 0.001 a	0.012 ± 0.0008 h
Magnesium (Mg) contents, g. kg^−1^			
Without Co			
Control	0.040 ± 0.001hi	0.031 ± 0.002 k	0.023 ± 0.003 l
Znfol	0.0.064 ± 0.002 f	0.056 ± 0.001 g	0.036 ± 0.005 ij
Zninj	0.053 ± 0.001 g	0.073 ± 0.001 e	0.039 ± 0.002 ij
With Co			
Control	0.078 ± 0.002 cde	0.071 ± 0.003 ef	0.029 ± 0.005 k
Znfol	0.086 ± 0.001 bc	0.090 ± 0.003 abc	0.041 ± 0.004 i
Zninj	0.076 ± 0.002 de	0.096 ± 0.002 a	0.044 ± 0.001 h
Sodium (Na) contents, g kg^−1^			
Without Co			
Control	0.787 ± 0.001 d	1.064 ± 0.0005 bc	1.188 ± 0.0005 d
Znfol	0.636 ± 0.001 e	0.497 ± 0.0003 g	1.068 ± 0.0007 e
Zninj	0.505 ± 0.001 fg	0.399 ± 0.0007 h	0.988 ± 0.008 fg
With Co			
Control	0.592 ± 0.001 efg	0.649 ± 0.0007 e	1.119 ± 0.0007 efg
Znfol	0.506 ± 0.001 fg	0.395 ± 0.001 h	1.023 ± 0.0006 fg
Zninj	0.519 ± 0.001 fg	0.381 ± 0.001 h	0.819 ± 0.0008 fg

The obtained values in the table are the average of the two growing seasons of 2021/2022.

Vertical bars represent ± standard error (SE) of the means. Bars on the top of the columns with different letters correspond to LSD are statistically significant at p ≤ 0.05. Different lowercase letters above error bars indicate statistically significant differences (p < 0.05). Abbreviations: Control (sprayed with pure water); Znfol (foliar chelated zinc application); Zninj (soil chelated zinc application); without Co (without cobalt sulfate application); with Co (with cobalt sulfate application, 7.5 mg L^−1^); (100%) GWR (applied 100% of gross irrigation water requirements); (80%) GWR (applied 80% of gross irrigation water requirements); (60%) GWR (applied 60% of gross irrigation water requirements).

#### Mg uptake

Likewise, by comparing the various treatments during both growing seasons, adopting the same examined treatments resulted in better Mg contents under (60% GWR) in the first season than the second, as can be seen in (Fig. 2B). While there were no significant differences between both seasons by adopting (100 and 80% GWR) in most treatments.

The results in (Table 6) showed a substantial fluctuation in peanut Mg content was seen during the growing seasons by adopting the various irrigation levels and using the examined applications. Whereas, by comparing the various irrigation levels in the control without applying Co application, adopting (100%) GWR resulted in better Mg content. Conversely, it was shown that the minimum Mg content could be achieved by adopting the (60%) GWR. It was found that by comparing the impacts of examined applications on Mg content, solitary application of Co attained higher Mg contents by adopting (100%) GWR. While under (80%) GWR, there were insignificant variations among the solitary applications of Zninj or Co for attaining better contents. Likewise, under (60%) GWR, it was found that the solitary applications of Znfol or Zninj significantly attained the same better Mg contents. Concerning the interaction, the obtained data indicated that by adopting (80%) GWR irrigation level, the highest Mg contents were observed by applying combined applications of (Zninj + Co), although that significantly equaled the adoption of (80%) GWR and applying combined application of (Znfol + Co).

#### Na uptake

Data in (Fig. 2C) showed that Na contents were statistically better in terms of yielding the better Na content in the second season than the first in most treatments especially under (80% GWR). While there were no significant differences between both seasons by adopting (100 and 60% GWR) in most treatments.

By comparing irrigation water levels in the control without applying Co application (Table 6), choosing (60%) GWR irrigation water levels, results in a significantly increases for Na contents in the peanut seeds compared to (80 and 60% GWR). In this sense, when adopting (80 and 60% GWR), Na content was increased than (100%) GWR by 25.8 and 33.6%, respectively. On the other side, by comparing the solitary applications of the examined applications, adopting (80%) GWR and applying the Zninj application resulted in the lowest Na contents, also the lowest contents under (100%) GWR were obtained by adopting solitary applications of Zninj or Co which were significantly equaled; while under (60%) GWR it could be significantly obtained by either adopting solitary applications of Znfol or Zninj. Furthermore, the obtained results showed that the combined applications of (Zninj + Co) under (80%) GWR were pronounced in equalizing the lowest recorded Na contents, although that significantly matched by adopting (80%) GWR and applying (Znfol + Co).

#### Fe uptake

**Figure 3:**
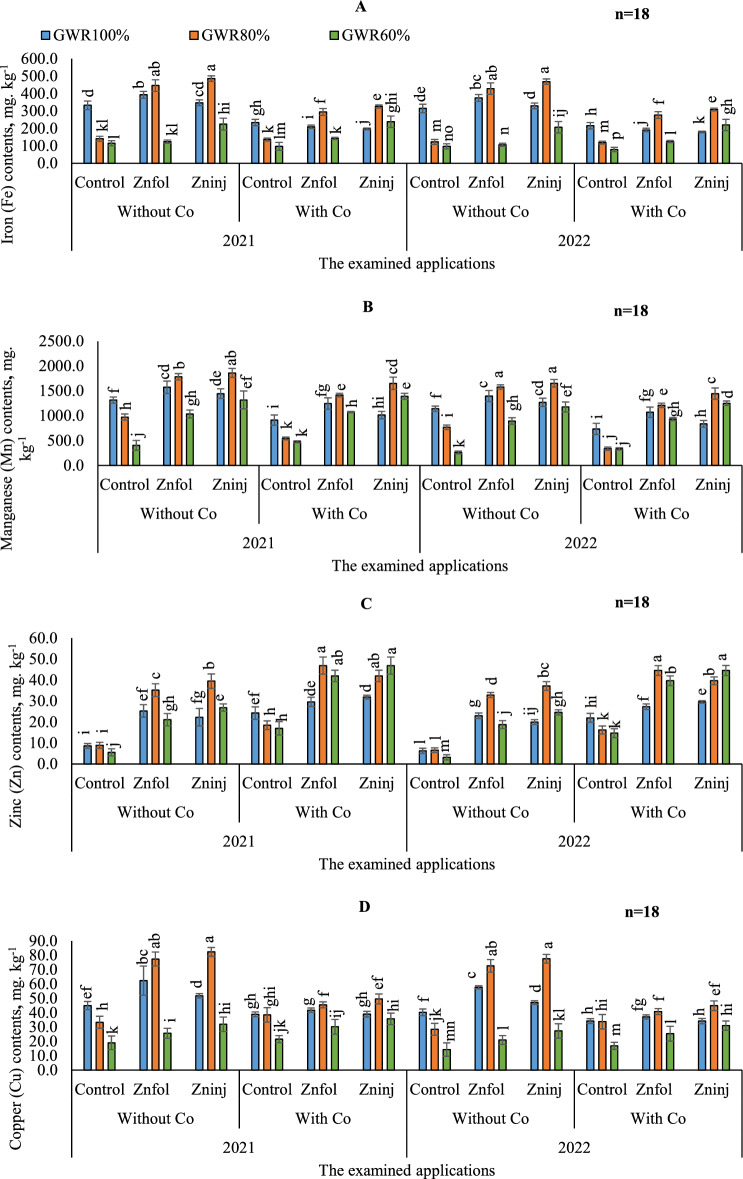
Influence of the separate or combined application of cobalt & chelated zinc under different irrigation levels during the growing seasons of 2021 and 2022 on: iron (**A**), manganese (**B**), zinc (**C**), and copper (**D**). Vertical bars represent ± standard error (SE) of the means. Values are means of n = 18 ± SE. Bars on the top of the columns with different letters correspond to LSD are statistically significant at p ≤ 0.05. Different lowercase letters above error bars indicate statistically significant differences (p < 0.05). Abbreviations: Control (sprayed with pure water); Znfol (foliar chelated zinc application); Zninj (soil chelated zinc application); without Co (without cobalt sulfate application); with Co (with cobalt sulfate application, 7.5 mg L^−1^); (100%) GWR (applied 100% of gross irrigation water requirements); (80%) GWR (applied 80% of gross irrigation water requirements); (60%) GWR (applied 60% of gross irrigation water requirements).

To compare the differences in Fe contents between the growing seasons, the results in (Fig. 3A) indicated that Fe contents were achieved better values under (60% GWR) in the first season compared to the second season in most examined applications.

Based on the results in (Table 7), executing (100%) GWR level under control treatment without applying Co application increased Fe contents in the peanut seeds. Interestingly, a negative effect was observed for Fe content when (80 and 60% GWR) irrigation levels were adopted under the same previous conditions. Although applying solitary applications of Znfol or Zninj, led to increased Fe content when adopting (80 and 60% GWR). Therefore, the obtained results indicated that treating stressed peanut plants with solitary soil applications of Zninj, could help plants overcome the negative impacts of water stress and improve Fe content. Generally, the results showed that by applying Co as a solitary or combined application, Fe contents recorded lower values than the non-Co treatment. In this sense, the adoption of solitary application of Znfol under (100%) GWR, improved Fe content compared to control (100%) GWR without applying Co application by 15.7%. While the solitary applications of Zninj were pronounced under (80% GWR) in attaining the highest increases in Fe contents by 127.6% than control (80%) GWR without applying Co.

**Table 7:** The impacts of adopting different irrigation water levels and applied chelated zinc as solitary and combined application with cobalt on the average iron, manganese, zinc, and copper.

Investigated parameters	Irrigation levels		
	(100%) GWR	(80%) GWR	(60%) GWR
Iron (Fe) contents, mg kg^−1^			
Without Co			
Control	323.9 ± 12.37 de	131.8 ± 7.09 j	105.8 ± 7.51 l
Znfol	384.0 ± 10.48 c	437.3 ± 16.8 b	114.8 ± 4.65 kl
Zninj	338.5 ± 8.33 d	477.3 ± 7.75 a	215.7 ± 16.72 gh
With Co			
Control	224.7 ± 9.17 g	127.2 ± 4.16 jk	87.7 ± 11.43 m
Znfol	200.3 ± 4.85 hi	285.5 ± 9.80 f	229.1 ± 16.40 g
Zninj	188.1 ± 2.37 i	317.5 ± 4.25 e	133.5 ± 3.24 j
Manganese (Mn) contents, mg kg^−1^			
Without Co			
Control	1228 ± 30.55 de	870 ± 31.42 gh	333 ± 50.37 j
Znfol	1485 ± 62.44 bc	1682 ± 31.86 ab	964 ± 39.08 f.
Zninj	1357 ± 48.56 de	1759 ± 45.33 a	1247 ± 90.3 de
With Co			
Control	823 ± 61.86 h	440 ± 13.73 i	404 ± 10.28 j
Znfol	1157 ± 57.51 ef	1312.8 ± 18.23 de	1009 ± 57.40 f.
Zninj	925 ± 36.18 fgh	1548 ± 113.2 bc	1321.5 ± 60.92 cdef
Zinc (Zn) contents, mg. kg^−1^			
Without Co			
Control	7.50 ± 0.56 j	7.70 ± 0.72 j	4.30 ± 0.85 j
Znfol	24.0 ± 1.48 fg	34.0 ± 1.5 c	19.9 ± 1.49 fg
Zninj	21.2 ± 2.08 gh	38.0 ± 1.7 b	25.7 ± 0.85 gh
With Co			
Control	23.0 ± 2.47 fgh	17.0 ± 1.05 i	15.9 ± 1.60 fgh
Znfol	28.5 ± 1.1 e	45.7 ± 1.37 ab	45.7 ± 2.05 e
Zninj	30.7 ± 0.44 de	40.8 ± 2.05 b	40.8 ± 1.37 de
Copper (Cu) contents, mg. kg^−1^			
Without Co			
Control	42.6 ± 1.40 e	30.9 ± 2.13 ij	27.1 ± 2.36 j
Znfol	60.1 ± 5.13 c	75.1 ± 2.45 b	23.3 ± 1.75 j
Zninj	49.5 ± 0.75 d	80.0 ± 1.53 a	29.6 ± 2.51 ij
With Co			
Control	36.5 ± 0.83 gh	36.2 ± 2.52 gh	16.6 ± 1.25 k
Znfol	39.5 ± 0.75 f.	43.0 ± 1.04 e	33.3 ± 2.0 hi
Zninj	36.6 ± 0.98 gh	47.3 ± 1.72 d	27.8 ± 2.57 j

The obtained values in the table are the average of the two growing seasons of 2021/2022.

Vertical bars represent ± standard error (SE) of the means. Bars on the top of the columns with different letters correspond to LSD are statistically significant at p ≤ 0.05. Different lowercase letters above error bars indicate statistically significant differences (p < 0.05). Abbreviations: Control (sprayed with pure water); Znfol (foliar chelated zinc application); Zninj (soil chelated zinc application); without Co (without cobalt sulfate application); with Co (with cobalt sulfate application, 7.5 mg L^−1^); (100%) GWR (applied 100% of gross irrigation water requirements); (80%) GWR (applied 80% of gross irrigation water requirements); (60%) GWR (applied 60% of gross irrigation water requirements).

#### Mn uptake

Likewise, as can be seen in (Fig. 3B**)**, by comparing the examined treatments throughout the growing seasons, adopting the examined (irrigation levels, Co, and Zn) resulted in better increases in Mn content under (60% GWR) in the first season than the second. While there were no significant differences between both seasons by adopting (100 and 80% GWR) in most treatments.

As can be seen in (Table 7), in the control treatment without applying Co application, Mn contents were decreased by 29.2% (80% GWR), compared with those under full-watered conditions (100%) GWR. On the other side, the solitary examined application resulted in fluctuating values under different irrigation levels, where it achieved matches maximum values by applying Znfol or Zninj under limited irrigation conditions (80% GWR) by 27.0 and 30.2%, respectively, compared with those under (100%) GWR without applying Co application. Similarly, Mn contents were attained the better values either by applying Znfol under full irrigation conditions (100% GWR) or Zninj under (60%) GWR, where it increased with Znfol by 17.3% compared with those under (100%) GWR and with Zninj by 1.5% compared with those under (100%) GWR, without applying Co application. Also, the results showed that the addition of distilled water applications under (60%) GWR attained a minimum value and decreased Mn content, which in the control treatment was 372.9% lower than the observed value in the (100%) GWR without applying Co application, and it was significantly equaled applying solitary Co applications under (60% GWR), which the reduction was 50.9% compared with those under (100%) GWR with applying Co application.

#### Zn uptake

The illustrated data in (Fig. 3C) showed that Zn contents were statistically better in terms of yielding the better Zn content in the first season than the second in most treatments.

The results in (Table 7) indicated that by comparing the various irrigation levels in the control without applying Co application, adopting (100 and 80% GWR) irrigation levels, resulted in the better Zn content. Conversely, it was shown that the minimum Zn content in peanut seeds could be attained by adopting (60%) GWR. It was found that by comparing the impacts of the examined applications on Zn content, solitary applications of (Znfol or Zninj, or Co) attained higher Zn contents by adopting (100%) GWR. While under (80 and 60% GWR) irrigation levels, the results indicated that the solitary applications of Zninj have the superiority for attaining better contents. Concerning the interaction, the obtained findings indicated that by adopting (80 and 60% GWR) GWR irrigation levels, the highest Zn contents were observed by applying combined applications of (Znfol + Co). Therefore, these results emphasize the importance of careful fertigation management, particularly when applying Zn applications under limited irrigation conditions. This approach catalyzes pronounced accumulation of Zn contents, resulting in improved peanut tolerance against water stress impacts.

#### Cu uptake

By comparing the various treatments during both growing seasons, adopting the examined treatments resulted in better Cu contents under (60% GWR) in the first season than the second in most treatments, as can be seen in (Fig. 3D). While there were no significant differences between both seasons by adopting (100 and 80% GWR) in most treatments.

By comparing irrigation water levels in the control without applying Co application (Table 7), choosing full-watered conditions (100%) GWR results in significant increases for Cu contents in the peanut seeds compared to (80 and 60% GWR). In this sense, when adopting (80 and 60% GWR), Cu content was decreased compared to (100%) GWR by 27.5 and 36.4%, respectively. On the other hand, by comparing the solitary applications of the examined applications, adopting (80%) GWR and applying the Zninj application resulted in the highest Cu contents. In this sense, the results demonstrated that it was increased by 17.3% compared with those under (80%) GWR without applying Co application. Furthermore, the obtained results indicated that the solitary application of Co under (60%) GWR, attained the lowest recorded value of Cu contents. In this sense, the results showed that relative to (100%) GWR and applying solitary application Co, Cu content decreased by 54.5%. These findings confirmed the need to avoid severe irrigation level exposure to peanuts when Co was adopted.

#### Soil pH

**Figure 4:**
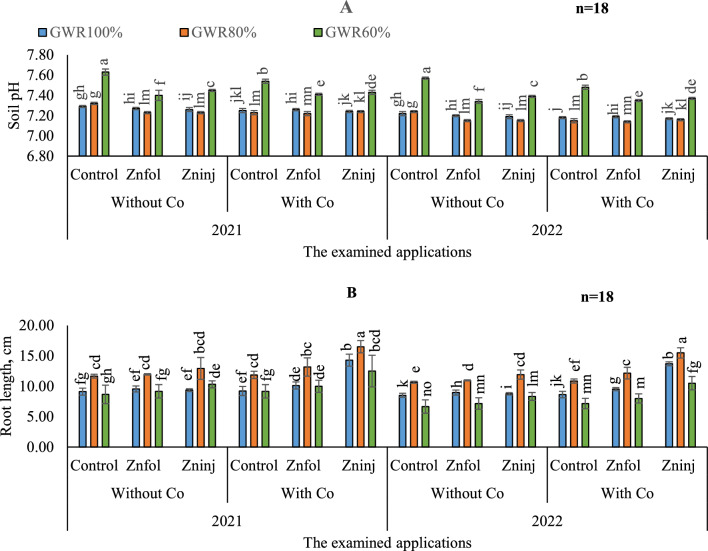
Influence of the separate or combined application of cobalt & chelated zinc under different irrigation levels during the growing seasons of 2021 and 2022 on: soil pH (**A**) and peanut root length (**B**). Vertical bars represent ± standard error (SE) of the means. Values are means of n = 18 ± SE. Bars on the top of the columns with different letters correspond to LSD are statistically significant at p ≤ 0.05. Different lowercase letters above error bars indicate statistically significant differences (p < 0.05). Abbreviations: Control (sprayed with pure water); Znfol (foliar chelated zinc application); Zninj (soil chelated zinc application); without Co (without cobalt sulfate application); with Co (with cobalt sulfate application, 7.5 mg L^−1^); (100%) GWR (applied 100% of gross irrigation water requirements); (80%) GWR (applied 80% of gross irrigation water requirements); (60%) GWR (applied 60% of gross irrigation water requirements).

By comparing soil pH differences between both growing seasons, no significant differences were found by adopting the same examined applications in most treatments, as can be observed in (Fig. 4A).

To maximize soil pH under different irrigation levels, it could be done by decreasing water irrigation amounts (Table 8). Where, based on the obtained results, it caused in increasing soil pH from 7.26 under (100%) GWR in control treatment without applying Co to 7.60 under (60%) GWR. And it caused in increasing soil pH up to 7.28 under (80%) GWR. When it comes to minimizing soil pH, any solitary applications under (100 and 80% GWR) irrigation levels, remains preferable. Similar pattern was also observed for the solitary application of Znfol under (60%) GWR. Concerning the interaction, the obtained data indicated that by adopting (80%) GWR irrigation level, the lowest soil pH values were observed by applying combined applications of (Znfol + Co), although that significantly equaled the adoption of (80%) GWR and applying solitary application of (Znfol or Zninj or Co).

**Table 8:** The impacts of adopting different irrigation water levels and applied chelated zinc as solitary and combined application with cobalt on the average soil pH, peanut root length, number of pods per plant, and number of seeds per plant.

Investigated parameters	Irrigation levels		
	(100%) GWR	(80%) GWR	(60%) GWR
Soil pH			
Without Co			
Control	7.3 ± 0.006 f.	7.32 ± 0.006 f.	7.6 ± 0.015 a
Znfol	7.24 ± 0.006 g	7.19 ± 0.006 g	7.37 ± 0.025 e
Zninj	7.22 ± 0.01 g	7.19 ± 0.006 g	7.42 ± 0.006 cd
With Co			
Control	7.21 ± 0.01 g	7.19 ± 0.012 g	7.51 ± 0.01 ab
Znfol	7.23 ± 0.006 g	7.18 ± 0.016 g	7.38 ± 0.006 e
Zninj	7.21 ± 0.006 g	7.20 ± 0.006 g	7.40 ± 0.01 d
Root length, cm			
Without Co			
Control	8.8 ± 0.29 fg	11.2 ± 0.15 de	7.7 ± 0.76 gh
Znfol	9.3 ± 0.25 f.	11.5 ± 0.58 d	8.2 ± 0.58 gh
Zninj	9.1 ± 0.12 fg	12.4 ± 0.93 cd	9.3 ± 0.28 fg
With Co			
Control	8.9 ± 0.38 fg	11.4 ± 0.32 de	8.2 ± 0.58 gh
Znfol	9.8 ± 0.29 f	12.7 ± 0.76 c	9.0 ± 0.5 fg
Zninj	14.0 ± 0.5 b	16.0 ± 0.5 a	11.5.5 ± 1.32 de
Number of pods, plant^−1^			
Without Co			
Control	59 ± 5.85 ab	42 ± 2.08 d	32 ± 2.51 g
Znfol	58 ± 2.64 b	39 ± 3.05 de	37 ± 1.15 ef
Zninj	56 ± 3.21 bc	41 ± 3.05 de	41 ± 1.15 de
With Co			
Control	56 ± 2.0 bc	41 ± 1.15 de	36 ± 3.46 efg
Znfol	51 ± 1.73 c	60 ± 4.5 ab	33 ± 2.3 g
Zninj	52 ± 2.0 bc	64 ± 2.08 a	41 ± 1.15 de
Number of seeds, plant^−1^			
Without Co			
Control	121 ± 10.81 abc	90 ± 8.8 de	83 ± 7.57 e
Znfol	123 ± 9.35 abc	85 ± 8.32 e	84 ± 2.0 e
Zninj	120 ± 10.0 bc	93 ± 10.06 de	79 ± 11.7 ef
With Co			
Control	114 ± 9.16 bc	94 ± 5.29 de	62 ± 11.5 ef
Znfol	104 ± 6.02 cd	118 ± 6.7 bc	63 ± 10.26 ef
Zninj	122 ± 13.51 abc	139 ± 6.42 a	85 ± 8.22 e

The obtained values in the table are the average of the two growing seasons of 2021/2022.

Vertical bars represent ± standard error (SE) of the means. Bars on the top of the columns with different letters correspond to LSD are statistically significant at p ≤ 0.05. Different lowercase letters above error bars indicate statistically significant differences (p < 0.05). Abbreviations: Control (sprayed with pure water); Znfol (foliar chelated zinc application); Zninj (soil chelated zinc application); without Co (without cobalt sulfate application); with Co (with cobalt sulfate application, 7.5 mg L^−1^); (100%) GWR (applied 100% of gross irrigation water requirements); (80%) GWR (applied 80% of gross irrigation water requirements); (60%) GWR (applied 60% of gross irrigation water requirements).

### The individual and interaction effects of irrigation levels, Co, and Zn applications on peanuts agronomic traits

#### Root length

As can be seen in (Fig. 4B**)**, by comparing both growing seasons, adopting the examined treatments resulted in the better increases in root length of peanut plants in the first season than the second.

By comparing irrigation water levels in the control without applying Co application (Table 8), adopting (80%) GWR irrigation water level results in significant increases in peanut root length compared to (80 and 60% GWR). On the other side, by comparing the solitary applications of the examined applications, adopting (80%) GWR and applying the Zninj application resulted in the tallest length root of peanuts; although that was significantly equaled the adoption of (80%) GWR and applying Co. Also, there were no marked difference was noted among the solitary examined applications under (60% GWR) irrigation level in attaining the shortest root’s length. Furthermore, the obtained results indicated that the combined applications of (Zninj + Co) under (80%) GWR were pronounced in achieving the tallest roots for peanuts.

#### The number of pods

**Figure 5:**
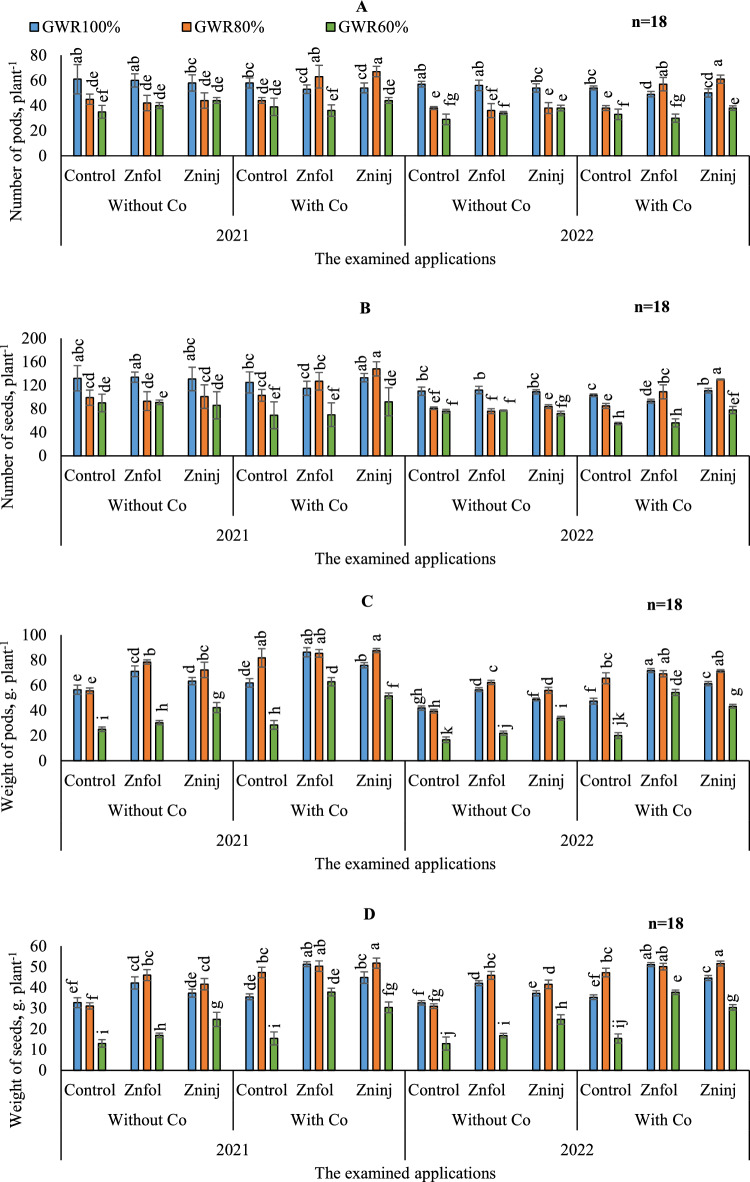
Influence of the separate or combined application of cobalt & chelated zinc under different irrigation levels during the growing seasons of 2021 and 2022 on: number of pods per plant (**A**), number of seeds per plant (**B**), weight of pods (**C**), and weight of seeds (**D**). Vertical bars represent ± standard error (SE) of the means. Values are means of n = 18 ± SE. Bars on the top of the columns with different letters correspond to LSD are statistically significant at p ≤ 0.05. Different lowercase letters above error bars indicate statistically significant differences (p < 0.05). Abbreviations: Control (sprayed with pure water); Znfol (foliar chelated zinc application); Zninj (soil chelated zinc application); without Co (without cobalt sulfate application); with Co (with cobalt sulfate application, 7.5 mg L^−1^); (100%) GWR (applied 100% of gross irrigation water requirements); (80%) GWR (applied 80% of gross irrigation water requirements); (60%) GWR (applied 60% of gross irrigation water requirements).

By comparing both growing seasons, there were no significant differences between growing seasons by adopting the same examined applications in most treatments, as can be seen in (Fig. 5A).

On the other hand, to maximize the number of pods per plant under different irrigation levels, it could be done by adopting full-watered conditions (100%) GWR (Table 8). Where, the obtained results indicated that it caused in increasing the number of pods from 32.0 under (60%) GWR in control treatment without applying Co to 59.0 under (100%) GWR, and it caused in increasing up to 42 under (80%) GWR. It was found that by comparing the impacts of examined applications on the number of pods, the results indicated that there was no marked difference noted between the solitary examined applications under the same irrigation levels and the same level in control treatment without applying Co, except for the (60%) GWR, where there was a marked difference noted compare to (60%) GWR in control treatment without applying Co. Concerning the interaction, the obtained results indicated that by adopting (80%) GWR irrigation level, the highest number of pods was observed by applying combined applications of (Znfol + Co), although that significantly equaled the adoption of (80%) GWR and applying combined applications of (Zninj + Co) or by applying the solitary application of distilled water applications (control) under (80%) GWR.

#### The number of seeds

Illustrated data in (Fig. 5B) showed that numbers of seeds per plant were statistically better in terms of yielding the better numbers of seeds under (80 and 60% GWR) in the first season than the second.

Similarly, the obtained results in (Table 8**)** indicated that adopting stressful irrigation levels (80 and 60% GWR) under without or with applying Co, led to decrease numbers of seeds per plant. However, applying solitary application of the examined applications under different irrigation levels interestingly didn’t lead to increase number of seeds significantly, when comparisons between the solitary examined applications and the same irrigation level in control treatment without applying Co, has been made. Overall, the highest number of seeds was obtained by adopting (100 and 80% GWR) irrigation levels and applying combined applications of (Zninj + Co), although that significantly equaled the adoption of (100%) GWR and applying solitary applications of Znfol or by applying solitary application of distilled water (control) under (100%) GWR.

#### The weight of the pods

To compare the differences in weight of pods between both growing seasons, it was observed that the better values were achieved in the first season compared to the second season under various examined applications in most treatments, as can be noticed in (Fig. 5C).

Likewise, the illustrated data in (Table 9) showed that by comparing the different irrigation levels in the control without applying Co application, adopting (100 and 80% GWR) resulted in improved weight of pods. Conversely, it was shown that a minimum weight of pods was attained by adopting (60%) GWR. Moreover, it was noticed that by comparing the impacts of examined applications on weight of pods, the higher values were attained by adopting solitary applications of Znfol under (100%) GWR, application of Co under (80%) GWR, and applications of Zninj under (60%) GWR. Concerning the interactions on the highest weight of pod values, the obtained results indicated that they were attained by applying combined applications of (Znfol + Co) under (100 and 80% GWR), although that significantly equaled the adoption of (80%) GWR and applying combined applications of (Zninj + Co) or by applying solitary application of Co under (80%) GWR.

**Table 9:** The impacts of adopting different irrigation water levels and applied chelated zinc as solitary and combined application with cobalt on the average weight of pods, weight of seeds, shoot fresh weight, peanut yield, and irrigation water use.

Investigated parameters	Irrigation levels		
	(100%) GWR	(80%) GWR	(60%) GWR
Weight of pods, plant^−1^			
Without Co			
Control	49.2 ± 1.8 f	47.5 ± 1.1 f	20.9 ± 6.9 h
Znfol	63.8 ± 2.1 c	70.4 ± 0.8 b	26.1 ± 0.8 h
Zninj	56.1 ± 1.4 e	64.1 ± 3.0 c	38.1 ± 2.0 g
With Co			
Control	54.7 ± 1.7 e	73.7 ± 3.6 ab	24.3 ± 1.7 h
Znfol	79.0 ± 1.8 a	77.3 ± 1.5 a	58.6 ± 1.6 de
Zninj	68.6 ± 1.0 b	79.4 ± 0.8 a	47.5 ± 1.0 f.
Weight of seeds, plant^−1^			
Without Co			
Control	32.6 ± 1.20 efg	31.1 ± 0.776 g	13.0 ± 4.4 j
Znfol	42.1 ± 1.45 d	45.9 ± 1.33 c	16.9 ± 0.55 ij
Zninj	37.2 ± 0.96 e	41.6 ± 1.36 d	24.6 ± 1.70 hi
With Co			
Control	35.4 ± 0.72 ef	47.3 ± 3.28 abc	15.4 ± 1.55 j
Znfol	51.2 ± 0.60 a	50.2 ± 1.26 ab	37.8 ± 0.95 def
Zninj	44.7 ± 1.37 c	51.7 ± 1.22 a	30.4 ± 1.3 fg
Shoot fresh weight, g m^−2^			
Without Co			
Control	2.128 ± 0.008 fg	2.228 ± 0.005 f	1.724 ± 0.009 h
Znfol	2.264 ± 0.005 f.	2.724 ± 0.002 e	2.148 ± 0.006 fg
Zninj	2.104 ± 0.004 g	2.68 ± 0.001 e	2.236 ± 0.004 f
With Co			
Control	2.624 ± 0.007 e	2.86 ± 0.005 cd	2.312 ± 0.004 f
Znfol	3.004 ± 0.010 bc	3.12 ± 0.005 b	2.988 ± 0.007 bc
Zninj	2.26 ± 0.006 f	3.332 ± 0.003 a	2.688 ± 0.015 e
Seeds yield, kg. ha^−1^			
Without Co			
Control	3916 ± 228.9 g	3728 ± 93.2 g	1556 ± 144.7 k
Znfol	5056 ± 66.09 d	5516 ± 159.8 c	2024 ± 174.9 j
Zninj	4468 ± 205.1 e	4988 ± 163.4 d	2952 ± 115.3 i
With Co			
Control	4248 ± 186.3 ef	5672 ± 193.7 c	1848 ± 86.53 j
Znfol	6144 ± 114.1 ab	6337 ± 151.9 a	3648 ± 72.9 h
Zninj	5364 ± 156.0 cd	5956 ± 147.5 b	4532 ± 164.9 e
Irrigation water use, kg. m^−3^			
Without Co			
Control	0.42 ± 0.02 h	0.49 ± 0.011 g	0.26 ± 0.05 ij
Znfol	0.55 ± 0.02 f	0.72 ± 0.02 bc	0.33 ± 0.01 i
Zninj	0.48 ± 0.01 g	0.65 ± 0.015 d	0.49 ± 0.03 g
With Co			
Control	0.46 ± 0.01 g	0.74 ± 0.04 bc	0.31 ± 0.03 ij
Znfol	0.67 ± 0.005 d	0.83 ± 0.02 a	0.60 ± 0.02 b
Zninj	0.58 ± 0.015 ef	0.78 ± 0.02 b	0.75 ± 0.02 bc

The obtained values in the table are the average of the two growing seasons of 2021/2022.

Vertical bars represent ± standard error (SE) of the means. Bars on the top of the columns with different letters correspond to LSD are statistically significant at p ≤ 0.05. Different lowercase letters above error bars indicate statistically significant differences (p < 0.05). Abbreviations: Control (sprayed with pure water); Znfol (foliar chelated zinc application); Zninj (soil chelated zinc application); without Co (without cobalt sulfate application); with Co (with cobalt sulfate application, 7.5 mg L^−1^); (100%) GWR (applied 100% of gross irrigation water requirements); (80%) GWR (applied 80% of gross irrigation water requirements); (60%) GWR (applied 60% of gross irrigation water requirements).

#### The weight of the seeds

As can be seen in (Fig. 5D), by comparing both growing seasons, there were no significant differences between both growing seasons by adopting the same examined applications under (100 and 80% GWR) irrigation levels in most treatments. While there were significant differences between both seasons by adopting (60%) GWR.

The results in (Table 9) showed that by comparing the irrigation levels in the control without applying Co application, adopting (100 and 80% GWR) irrigation levels resulted in a better weight of seeds value. Conversely, it was shown that the minimum weight of seeds value was attained by adopting (60%) GWR. It was found that by comparing the effects of examined applications on the weight of seeds compared to control treatment without applying Co, the solitary application of Co attained the higher weight of seeds value by adopting (80%) GWR irrigation level. While under (100%) GWR irrigation level, the results indicated that the solitary applications of Znfol have superiority for attaining better value. Likewise, under (60% GWR) irrigation level, the solitary applications of Zninj have superiority for attaining better value. On the other side, the obtained findings indicated that by adopting (100 and 80% GWR), the highest weight of seed values were observed by applying combined applications of (Znfol + Co), although that significantly equaled the adoption of (80%) GWR and applying combined applications of (Zninj + Co) or by applying solitary application of Co under (80%) GWR.

#### Shoot fresh weight

**Figure 6:**
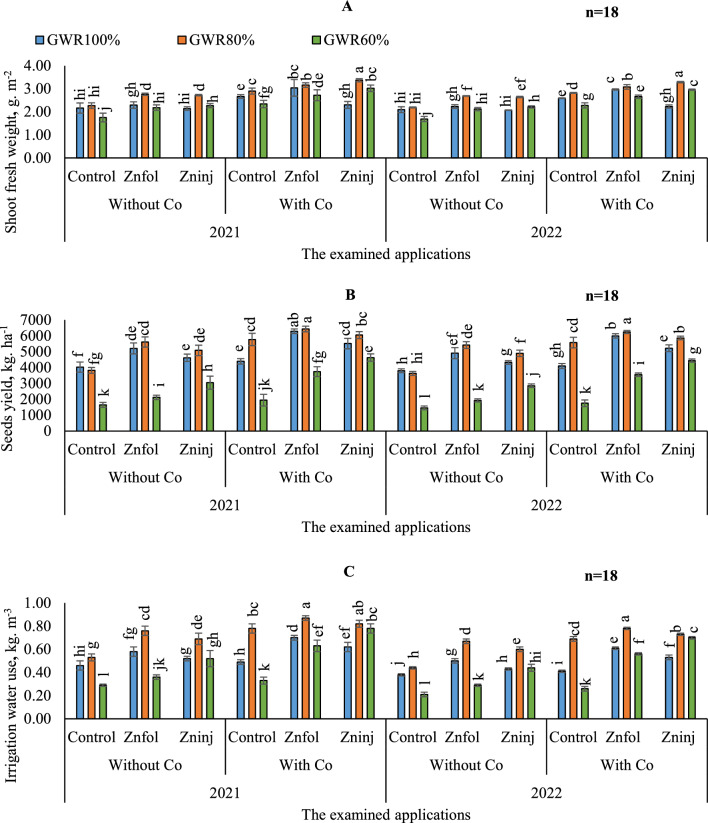
Influence of the separate or combined application of cobalt & chelated zinc under different irrigation levels during the growing seasons of 2021 and 2022 on: shoot fresh weight (**A**), peanut yield (**B**) and irrigation water use (**C**). Vertical bars represent ± standard error (SE) of the means. Values are means of n = 18 ± SE. Bars on the top of the columns with different letters correspond to LSD are statistically significant at p ≤ 0.05. Different lowercase letters above error bars indicate statistically significant differences (p < 0.05). Abbreviations: Control (sprayed with pure water); Znfol (foliar chelated zinc application); Zninj (soil chelated zinc application); without Co (without cobalt sulfate application); with Co (with cobalt sulfate application, 7.5 mg L^−1^); (100%) GWR (applied 100% of gross irrigation water requirements); (80%) GWR (applied 80% of gross irrigation water requirements); (60%) GWR (applied 60% of gross irrigation water requirements).

By comparing the various treatments during both growing seasons, there were no significant differences between both growing seasons by adopting the same examined applications in most treatments, as can be seen in (Fig. 6A).

The obtained results in (Table 9) showed that by comparing the various irrigation levels in the control without applying Co application, adopting (100 and 80% GWR), resulted in better shoot fresh weight values. Conversely, it was shown that the minimum shoot fresh weight value was attained by adopting (60%) GWR. It was found that by comparing the effects of examined applications on shoot fresh weight, solitary application of Co attained higher shoot fresh weight value by adopting (100 and 80% GWR) irrigation levels. But no marked difference was observed among the sole examined applications under (60% GWR) irrigation level. Additionally, the obtained findings indicated that by adopting (80%) GWR irrigation level, the highest shoot fresh weight values were observed by applying combined applications of (Zninj + Co).

### The individual and interaction impacts of the examined irrigation levels, Co, and Zn applications on

#### Peanuts yield

Based on the results of variance analysis (Table 4), the individual and interaction effects of examined irrigation levels, Co, and Zn obviously affected peanut yield.

To compare the differences in peanut yield between both growing seasons, it was found that the better values were achieved in the first season compared to the second season in most examined applications, as can be observed in (Fig. 6B). Compared to the examined irrigation levels in control treatment without any auxiliary applications, peanut yield was decreased by adopting (60%) GWR irrigation level compared to (100%) GWR by 60.3%, but, no marked difference was observed under (80%) GWR (Table 9). Therefore, treating stressed plants (80 and 60% GWR) with auxiliary applications, could help peanut plants overcome the negative impacts of water stress and improve yield under these conditions. In this sense, it was found that by comparing the impacts of examined applications on yield under the same irrigation level, the results demonstrated that the solitary application of Znfol attained higher value and increased yield by 22.6% compared to (100%) GWR in control treatment without applying Co; when (100%) GWR irrigation level was adopted. Likewise, under (80%) GWR, the results indicated that the solitary applications of Znfol or Co were significantly equaled, and they have superiority for attaining better increases by 29.0 and 31.0%, respectively. A similar pattern was also noted under (60% GWR) irrigation level, the solitary applications of Zninj have superiority for attaining the better value. Concerning the interaction, the obtained results showed that by adopting (100 and 80% GWR) irrigation levels, the highest yield was observed by applying combined applications of (Znfol + Co).

##### IWUE

Similarly with peanut yield, illustrated data in (Fig. 6C) showed that IWUE was statistically better in terms of yielding the values in the first season than the second in most examined treatments.

Generally, the obtained results in (Table 9), showed that increasing IWUE under different irrigation levels could be done by adopting (80%) GWR. Where, compared to control (100%) GWR without applying Co, the IWUE was increased with the adoption of (80%) GWR by 14.3% and decreased by 38.1% with the adoption of (60%) GWR. On the other side, the adoption of solitary applications of Znfol under (100% GWR), improved IWUE compared to control (100%) GWR without applying Co application by 23.6%. While the solitary applications of Zninj or Co were pronounced under (80% GWR) for attaining better increases in IWUE by 31.9 and 33.8%, than control (80%) GWR without applying Co, respectively. Likewise, the adoption of solitary applications of Zninj under (60% GWR), increased IWUE compared to control (60%) GWR without applying Co application by 46.9%. On the other hand, the gained results showed that the maximum increase of IWUE, was observed by adopting combined applications of (Znfol + Co) under (80%) GWR irrigation level.

## Discussion

Water stress is a significant challenge to global agricultural production, necessitating the adoption of specific methodologies to determine suitable water regimes for different crops^6,9^.

### Impacts of various irrigation levels on macronutrient uptake, physiological status, and growth

In the present study, subjecting peanut crops to water stress at (60%) of the GWR resulted in a critical reduction of absorbed or stored water within tissues due to decreased root length, and increased transpiration rates, leading to higher water loss from the aerial parts faster than the roots could absorb^52^. Additionally, adopting the (60%) GWR, along with increased soil evaporation due to higher temperatures (Table 1), decreased the amount of water required to leach alkaline salts, resulting in an increase in soil surface pH**.** Consequently, this affected root efficiency, leading to reduced solubility and uptake of macronutrients, especially P, which is known to enhance root growth, architecture, and efficacy^55–57^. Although plants under such conditions tend to increase root exudates as a tool to increase nutrient bioavailability^58–60^, using this trend alone did not seem effective under these circumstances. The study found significant reductions in all macronutrients, except for Na. The decline in root efficiency can be attributed to (A) reduced root absorbent efficiency either due to (1) the decreased available water, which reduces their penetration ability^61^, or (2) increasing Na concentrations around roots, resulting in Na toxicity and competition against other nutrients for root sites^62^. (B) increasing nutrient fixation processes compared to root exudate amounts. Regardless, declining root efficiency along with increasing soil pH led to pronounced reductions in micronutrient uptake, as pH changes severely affect nutrient uptake^63^. As a consequence, the physiological and biological processes within plants were affected^25^, ultimately resulting in reduced agronomic traits and yield of peanuts. On the other hand, increasing irrigation levels had positive impacts on peanut traits, yield, and IWUE, with the most pronounced impact observed when adopting (80%) GWR compared to (100%) GWR. These findings were attributed to the increased water quantities, which improved root length and absorption efficiency, leading to increased macronutrient uptake. Additionally, root exudates decreased soil pH, thereby increasing micronutrient uptake and reflecting in enhanced yield and IWUE. However, the study was unable to explain why both (100&80%) GWR irrigation levels resulted in equal yields, despite better concentrations of macronutrients and micronutrients under the (100%) GWR irrigation level. Other studies^5,64^ have observed that applying full irrigation levels did not always result in high chlorophyll content in peanut leaves and (excessive irrigation water and N uptake) could lead to diseases in peanut leaves, negatively affecting yield. Nonetheless, this study was unable to confirm these aspects due to limitations in measuring chlorophyll and diseases under the current experimental conditions.

### Optimum Zn application method under various irrigation levels

Applying separate amounts of Zninj under (60%) GWR irrigation level was more effective than Znfol, consistent with previous study^25^. Under (60%) GWR, plants tend to reduce some activities, including transpiration rates, by closing stomata as a protective mechanism against water stress. In this situation, roots play a crucial role in controlling plant activities. Applying Zninj has numerous benefits, including improved nutrient uptake, leading to enhanced crop yield, which aligns with similar findings in previous studies^65,66^. Conversely, under (100 & 80%) GWR levels, many stomata were open due to adequate soil moisture, resulting in increased transpiration rates^67,68^. This caused an imbalance in nutrient uptake activities between the most active plant aerial parts and roots, consistent with previous studies^5,23^. The current study’s findings suggest that under similar growth conditions (100 & 80%) GWR, applying foliar applications of Znfol to peanut plants has desirable effects. This application method increases Zn absorption and compensates for root inefficiency, leading to increased nutrient absorption and improved potential for yield and IWUE.

### Impacts of Co application under various irrigation levels

Under (60%) GWR irrigation level, peanut plants experienced critical reduction in soil moisture, leading to decreased nutrient uptake, particularly for P, which promotes root growth and architecture^56^, causing a decrease in root system penetration. When Co was applied under these conditions, its molecules accumulated near or in the roots, making the roots more susceptible to water availability and Co concentrations^69^. Additionally, the reduction in soil moisture decreased root activities and exudates, resulting in decreases in (N, P, Fe, Mn and Cu) uptake, and an increase in soil pH. The higher values of soil pH and Co led to antagonistic relations between Co and Fe, Mn, and Cu under these conditions. This was partly due to reduced nutrient availability under higher pH and competition between these nutrients for transport sites in the root, leading to a reduction in photosynthesis, consistent with previous findings^21,22^. These detrimental effects ultimately reflected in the reduction of peanut agronomic traits, peanut yield, and IWUE, which align with previous studies^70–72^. Dang et al.^73^ also highlighted that plant growth and metabolism vary based on the concentration and state of Co in the rhizosphere and soil. Hence, previous studies^5,23^ demonstrated that Co benefits are correlated with the irrigation level used. Hence, supplying stressful peanut plants with sole applications of Co proved to be a harmful technique, resulting in adverse impacts on yield and IWUE.

To resolve the issues caused by sole Co applications to stressful peanut plants, two approaches can be taken. (A) Using a moderate irrigation level (80%) GWR, where root length, exudates, and absorption efficiency improve, leading to decreased soil pH and enhanced macronutrient and micronutrient uptake, resulting in increased yield and IWUE. (B) Supplying plants with Zninj application in combination with Co under either (80%) or (60%) GWR levels. Under these conditions, Zninj can form chelating components with Fe and other micronutrients, preventing their fixation induced by P or Ca, which enhances their ability to compete with Co for capture sites on peanut roots, ultimately resulting in the observed improvements in the study, consistent with previous finding Zanin et al.^74^.

### Optimum combined applications of Co and Zn under various irrigation levels

The previous findings have clarified the individual impacts of the examined applications under different irrigation levels. However, when considering combined applications, the results indicated that achieving higher yield and IWUE depended on the irrigation levels and the method of Zn application used in combination with Co. Under (60%) GWR level, the benefits of applying combined Co + Zninj applications, as discussed earlier, resulted in the best growth performance, yield, and IWUE under these conditions. On the other hand, the highest yield and IWUE values were recorded by applying Co + Znfol under (100&80%) GWR levels. The study attributed these results to the benefits of adopting (100&80%) GWR levels, which increased root length and efficiency, leading to an increase in root exudates and a decrease in soil pH, resulting in notable improvements in water and nutrient absorption rates from the soil rhizosphere. This is supported by previous studies^75–77^. Additionally, under these conditions, a significant number of stomata were open, and applying Znfol allowed Zn molecules to penetrate leaves stomata, improving nutrient contents directly and quickly and compensating for the lack of nutrient absorption by the root. Furthermore, Znfol led to better improvements in the absorption of k, Fe and Cu compared to Zninj, which promoted physiological processes. Zn plays a vital role in many enzymes, biochemistry, metabolism^78^, water relations^79,80^, membrane stability^81^ and stomatal regulation^82,83^. This led to improved plant tolerance to abiotic stress, decreased water losses from the plant, and improved shoot fresh weight, weight of pods, and weight of seeds. Similarly, Co application under (100&80%) GWR improved transpiration, stomatal conduction, nodulation, N fixation, and assimilation in peanut plants, in agreement with previous studies^20,22^. The combined applications of Co + Znfol contributed to decreasing the pH value, due to the benefits of Co application on microorganism activity. The findings indicate that the combination improved the absorption of (N, P, Mg and Zn) nutrients, which led to an improvement in the number, weight of pods weight of seeds. Previous studies have also reported that foliar application of Zn affects yield and agronomic traits, enhancing the number of pods per plant Banks^84^. These results align with studies that suggest Co and Zn applications can enhance plant biomass, enzymatic actions, and physiological and chemical processes, leading to improved transpiration rates^85,86^. Therefore, applying the combined applications of Co + Znfol improved plant performance, growth, and nutrient uptake. Although better yield increases were observed under (80%) GWR level than (100%) GWR, resulting in the highest IWUE value, the study recognizes the need for more information on the impacts of Co and Zn (Znfol or Zninj) on soil physiochemical properties and their dynamic influences on nutrient status in roots, shoots, and seeds under different irrigation schemes. Such data would contribute to a more comprehensive understanding of peanut plant growth, yield, and IWUE.

## Conclusion

The current study highlights that peanut plants are negatively impacted by severe water stress. To mitigate these effects, supplying stressed plants with auxiliary combined applications of cobalt and chelated zinc can increase nutrient absorption, yield, and water use efficiency. The findings suggest that peanut tolerance to water stress can be enhanced by applying sole soil applications of chelated zinc under 60% of the gross water requirements and solitary foliar applications of chelated zinc under 80&100% levels. However, the combined applications with cobalt under these irrigation levels showed even more positive increases in yield. Further research is needed to understand the impacts of cobalt and zinc (Znfol or Zninj) on soil physiochemical properties and their dynamic effects on nutrient status in roots, shoots, and seeds under different irrigation levels. This deeper understanding will provide a basis for developing management strategies to improve peanut plant growth, yield, and irrigation water use efficiency in arid areas. Based on the findings, it is recommended to apply combined applications of foliar chelated zinc in combination with cobalt under 80% of the gross irrigation water requirements for peanuts as auxiliary applications to stressed plants. This approach can enhance nutrient uptake and yield and ameliorate the impact of water stress, leading to improved water use efficiency in peanut crops.

### Supplementary Information

Supplementary Figure S1.

## Data Availability

The presented datasets during the current study available from the corresponding author on reasonable request.

## Abbreviations

GWR: Gross water requirements
Znfol: Foliar applications of zinc
Zninj: Zinc applied injection by irrigation systems
ETcrop: Crop evapotranspiration
IWUE: Irrigation water use efficiency
Co: Cobalt
