# Supplementary material for: Impacts of cobalt and zinc on improving peanuts nutrient uptake, yield and irrigation water use efficiency under different irrigation levels
Source: Sci Rep. 2024 Mar 26;14:7188. doi: 10.1038/s41598-024-56898-2 (PMC10966013; doi:10.1038/s41598-024-56898-2)
Supplement: Supplementary file 1 — Supplementary Figure S1. [file 41598_2024_56898_MOESM1_ESM.pdf]

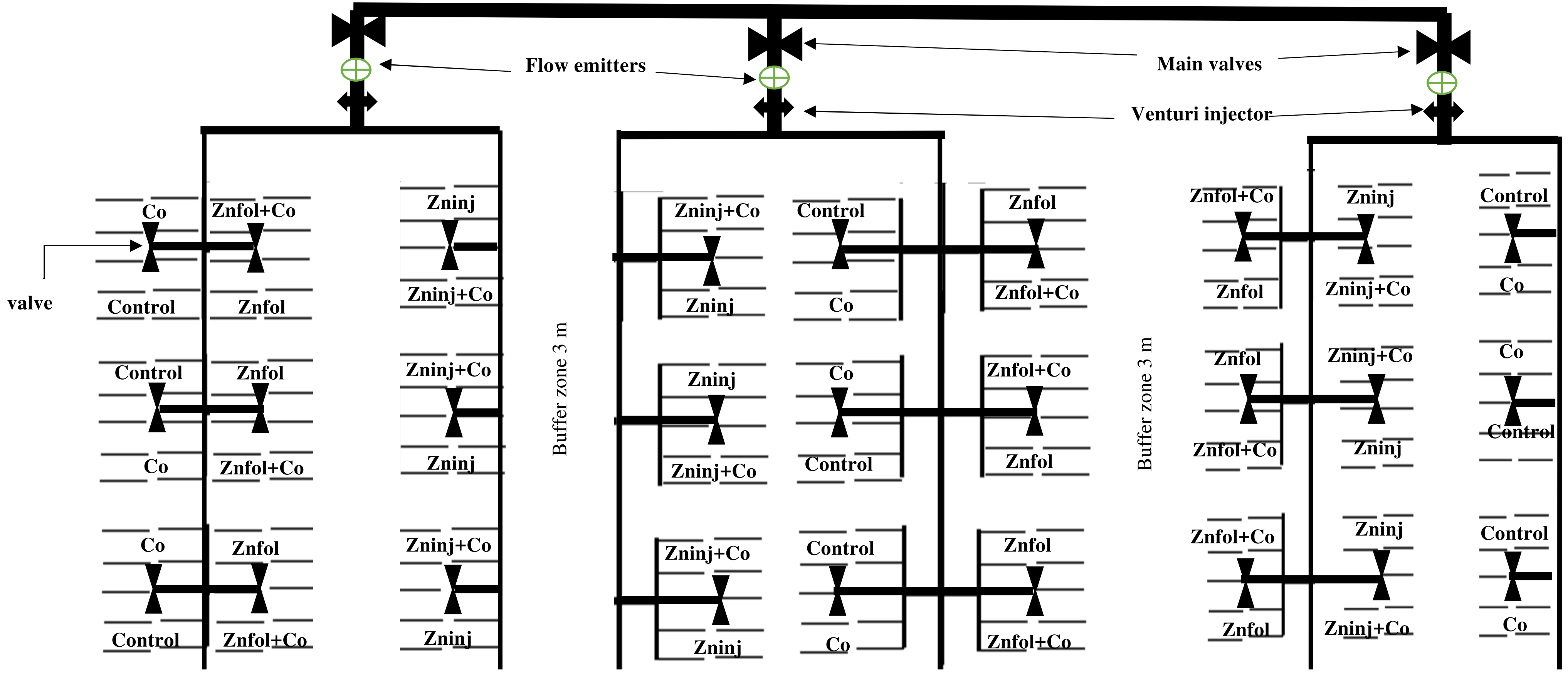

60% of peanuts gross water requirement

80% of peanuts gross water requirement

100% of peanuts gross water requirement

Fig. S1 Experiment layout with a distribution of irrigation levels and examined applications. Abbreviations: Control (sprayed with pure water); Znfol (foliar chelated zinc application); Zninj (soil chelated zinc application); without Co (without cobalt sulfate application); with Co (with cobalt sulfate application, 7.5 mg L^{-1}).
